# Experimental Proof of Principle of 3D-Printed Microfluidic Benthic Microbial Fuel Cells (MBMFCs) with Inbuilt Biocompatible Carbon-Fiber Electrodes

**DOI:** 10.3390/mi15070870

**Published:** 2024-06-30

**Authors:** Terak Hornik, Maxwell Terry, Michael Krause, Jeffrey K. Catterlin, Kevin L. Joiner, Samuel Aragon, Angelica Sarmiento, Yolanda Meriah Arias-Thode, Emil P. Kartalov

**Affiliations:** 1 Physics Department, Naval Postgraduate School, 1 University Circle, Monterey, CA 93943, USA; tbhornik@gmail.com (T.H.); maxwell.terry@nps.edu (M.T.); jkcatter@nps.edu (J.K.C.); 2 MOVES Institute, Naval Postgraduate School, 1 University Circle, Monterey, CA 93943, USA; michael.krause@nps.edu; 3 Naval Information Warfare Center, San Diego, CA 92152, USA; kevin.l.joiner.civ@us.navy.mil (K.L.J.); sjaragon@sdsu.edu (S.A.); angelica.v.sarmiento.civ@us.navy.mil (A.S.); yolanda.m.arias-thode.civ@us.navy.mil (Y.M.A.-T.)

**Keywords:** microbial fuel cell, benthic microbial fuel cell, microfluidic, 3D printing, electrogenic bacteria, carbon fiber, biocompatible, electrical, wiring

## Abstract

Microbial fuel cells (MFCs) represent a promising avenue for sustainable energy production by harnessing the metabolic activity of microorganisms. In this study, a novel design of MFC—a Microfluidic Benthic Microbial Fuel Cell (MBMFC)—was developed, fabricated, and tested to evaluate its electrical energy generation. The design focused on balancing microfluidic architecture and wiring procedures with microbial community dynamics to maximize power output and allow for upscaling and thus practical implementation. The testing phase involved experimentation to evaluate the performance of the MBMFC. Microbial feedstock was varied to assess its impact on power generation. The designed MBMFC represents a promising advancement in the field of bioenergy generation. By integrating innovative design principles with advanced fabrication techniques, this study demonstrates a systematic approach to optimizing MFC performance for sustainable and clean energy production.

## 1. Introduction

Microbial fuel cells (MFCs) represent a method of generating electrical power via microbial processes by the connection of an anaerobic anode to an aerobic cathode [1,2,3,4]. These processes generally involve anaerobic bacteria oxidizing organic matter and subsequently producing electrical current [5,6]. The ocean floor ecosystem contains diverse and abundant microbial activity [7,8]. A Benthic MFC (BMFC) functions similarly to an MFC with the added specification of making use of bacteria that naturally occupy this ecological niche in a marine environment [2].

Some anaerobic bacteria are able to expel their terminal electrons to the external environment; these are exoelectrogens [9]. It is these expelled electrons that are captured by the anode of a BMFC. This is performed via a conductive capture anode connected to a nearby cathode by a resistive load, thus obtaining usable electrical power from the bacteria. Typical voltage ranges for this arrangement span from 0.2 to 1 V [10,11,12,13,14,15]. This technique is applied in practice with applications such as low-draw sensors [11,12,13].

Typical BMFCs have been shown to produce low amounts of power in the range of 3–40 mW/m^2^ in situ [10,12,13,14,15,16] and as high as 1 W/m^2^ in laboratory environments with optimal conditions including nutrition being provided [17,18,19,20,21]. Regarding the in situ BMFCs, there have been various attempts to improve the resulting power densities via various strategies such as altering the cathode (e.g., increasing the size) [22,23,24,25], altering the anode [1,23,26], altering both electrodes [23,27,28], changing the electrode spacing, coating the electrodes in cerium [27], using carbon felt as the anode material [29], using organic additives such as acetate [4,10,28,30,31,32,33], and supplying alternative food sources like chitin [16].

Alternative optimization approaches involve bacterial concentration, anode geometry, or the minimization of the distance from the cells to the anode [28]. Optimizing bacterial concentration would intuitively increase power production proportionally. However, there are diminishing returns (or even decreases) associated with this approach due to the ecological constraints on the bacterial communities [34,35].

Due to the size of bacteria, they live in a world dominated by viscous forces, characterized by low Reynolds numbers, typically values less than 1 [36,37]. The environment created within microfluidic devices can also be characterized by low Reynolds numbers which matches the needs of the microbes [38,39]. Making use of these low Reynolds number regimes is common practice in fields such as biotechnology [40], cell culture work [41], biomedical diagnostic tools [42], and embedded electric measurements [43]. Microscale MFCs are generating lots of interest due to the potential benefits to capture efficiency and the continuing efforts to maximize power density [44,45,46,47].

A united approach, joining microfluidics chips and living microbial systems, can be used to improve the characterization of Microfluidic BMFC (MBMFC) relevant microbial responses [40,48]. This can be performed by depositing a metal electrode on glass, then using that surface as the substrate for an elastomer microfluidic chip [43]. Elastomer microfluidic chips such as these boast key benefits such as biocompatibility, fluidic control, visibility, and critically, the minimization of the distance between the bacteria and the anode [28,38].

This approach was successfully used to constrain the bacteria to be within 90 μm of the capturing electrode in a proof-of-concept experiment yielding an increase in generated power by roughly a factor of four [49]. This showed that the shortening of distance between the electron emission and capture significantly improved the power production capabilities of MBMFCs. This was performed with a PDMS (polydimethylsiloxane) cast as the physical structure of the microfluidic component. The capturing electrode was chromium in a fractal pattern. The test devices were prepared with a single-input single-output microfluidic design with dendritic branching [49]. They were placed in sediment collected from the San Diego Bay. Steady-state power densities as high as 80 mW/m^2^ were recorded [49]. It follows that further confinement of the bacteria could further increase the performance.

However, these test devices were difficult and time-consuming to fabricate, thus presenting the problem of how to upscale effectively. Depending on the applications, thousands or even tens of thousands of these test devices would need to be implemented together for practical use. Even if they are easy to produce in small numbers, the cost of producing enough of them and assembling them would still be a significant deterrent. Thus, a pathway is needed to allow for efficient upscaling. Three-dimensional printing offers just such a pathway to upscale microfluidic devices such as these [50].

The 3D printing of embedded negative features, such as three-dimensional networks of microfluidic channels, requires the removal of the sacrificial support material inherent to 3D printing. We previously addressed this via a clearing methodology developed specifically for this application [51]. This technique is based on the combination of vibration, heating, and chemicals injected into the microchannels. The methodology works reliably when the microchannels are wider than 200 μm across [51].

Another key development leading to the practical use of 3D printing in microfluidic applications like these is predictable manufacturing. Using PolyJet technology [52], we have highly reproducible fabrication. Thus, planning is now possible around the systematic recurrent discrepancies between dimensions on a CAD model and their analog in an associated 3D print [52]. Reproducibility and the consequent predictability are critical for industrial applications as well as further research and development.

The next hurdle to address was embedding electrical wiring in the microfluidic network. We initially approached this task via a self-assembly technique applied to square microfluidic channels with flanges on the sides [53]. After removing the sacrificial material [51], the microchannel was filled with a hydrophobic fluid. Next, the hydrophobic fluid was flushed out of the central area of the microchannel with a hydrophilic fluid at moderate pressure, while the hydrophobic fluid in the flanges remained in place due to higher surface tension and fluidic resistance in the flanges [53]. The next step was for the hydrophobic fluid to be conductive, e.g., nanoparticle-spiked resin to be cured in position post-self-assembly. This could then act as a pair of wires that run parallel to the microchannel. With a square channel 200 μm across, this achieved roughly the same maximal spacing between any bacterium and the nearest electrode as the one that previously increased the power output by a factor of four [49,53]. This approach using resin spiked with conductive nanoparticles was hindered by the mixed fluid being too viscous to feed into the microchannel.

An alternative method was developed based on deposition of conductive particles directly inside the microchannels. This led to Carbon Nano Fiber (CNF) distributed wiring throughout the entire volume of a microchannel [54]. Importantly, carbon is a biocompatible electrode material. Additionally, this approach maintains some key benefits of that self-assembly method: access is only needed through a fluidic inlet; the wiring is performed via fluidic injection, and can thus be potentially automated in the future; and acceptable conductivity is achievable.

In this scheme, the capturing anode is distributed throughout the volume of the microchannels. This further minimizes the distance from an emitted electron to the nearest electrode, as the bacteria and wiring occupy portions of the same physical space. This scheme provides a definite improvement to the separation distance compared to the preceding work [49]. With these developments, the key fundamental prerequisite developments such as fabrication and wiring methods have now been addressed.

Herein, we present a monolithically 3D-printed microfluidic device containing a binary branching microfluidic network with embedded CNF wiring as the biocompatible volumetrically distributed electrode. The typical achieved electric conductivity per dry microchannel is 4 S/m. This device is an important step in the further development of MBMFCs. In the future, this can be further developed and optimized before being used as an integral unit element within modular macro-scale MBMFCs. As such, this represents a major step in the advancement of this technology and carries strong promise for the future of MBMFCs.

The potential applications include energy at the sea floor or near the sea floor. The end goal is to stack these small-scale units to make microbial condominiums to eventually obtain 1 W of power at the sea floor.

## 2. Materials and Methods

***Chip Architecture***. The test chips discussed herein can be seen in Figure 1. We call them `comb’ chips due to their visual resemblance to a hair comb. They are designed within an outer frame defined by a rectangular prism of dimensions 100 × 70 × 5 mm. The bottom portion of the comb chips houses 32 identical microfluidic channels (0.8 × 0.8 mm cross section) that form the fluidic outlets. The upper portion is dedicated to a dendritic branching structure that connects the single common inlet port to all the 32 outlets. The total internal volume of the microfluidic network as defined by SOLIDWORKS is 1040 mm^3^.

***Chip Fabrication***. These chips are designed in SOLIDWORKS 2022 (see Figure 1), then converted into STL files for compatibility with our 3D printer: Stratasys Objet500 Connex 1 (Stratasys Ltd., Rehovot, Israel). The models are laid flat on the print bed and oriented to keep the microchannels in the bottom portion of the design parallel to the primary axis. This maximizes the print quality [52]. Devices are printed in batches to produce a total of 10. The material used for the main structure of the chip is VeroClear-RGD810, a clear hard resin chosen for its optical clarity, chemical resilience, and mechanical rigidity. Additional materials used during printing are SUP706B for the sacrificial material, and AGILUS30 BLACK FLX985 for the labels.

***Support Material Removal***. After printing, the chips are put through our previously published clearing procedure [51]. Briefly, this process includes the flushing of channels and sonication of the chips in a 10% NaOH aqueous solution as well as heating at 80 °C. They are then cleaned of any residual chemicals and carefully inspected. This procedure successfully clears each of the 320 total channels.

***Chip Wiring***. Once the test chips are cleared of support material, the next step in the process is electrical wiring. This is accomplished by depositing CNFs throughout the volume of the microchannels [54]. Briefly, the CNFs are implanted by injecting a solution of CNFs in isopropyl alcohol into the entire internal volume of the chips. Next, the chips are heated to evaporate the alcohol while leaving the CNFs in place. This process is iterated 10 times, incrementally improving conductivity. Lastly, a naked copper wire is inserted in parallel to a plastic Luer-Stub adapter into the primary inlet and epoxied in place. This allows for simultaneous fluidic and electrical access to the internal network of microchannels via the primary inlet. See Test and Evaluation, MBMFC construction for a wiring description during the test and evaluation phase.

***Electrical Characterization***. All electrical measurements are taken via a benchtop source meter (Model 2400, 119 Keithley Instruments LLC, Cleveland, OH, USA). For each datapoint, the instrument takes 100 sample measurements automatically and calculates the mean value and the standard deviation. These are then recorded as the corresponding measured value and its error bar, respectively. All electrical measurements are taken in this way.

**Figure 4:**
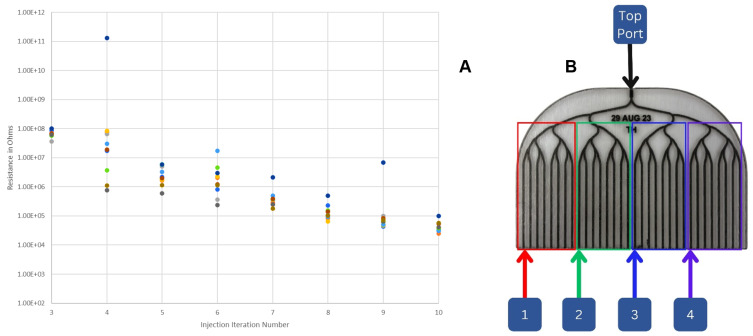
The average resistance of each comb device as a function of injection number and definitions of representative subsets. (**A**) shows that the average resistance value is determined by averaging the measurements from the inlet to 4 outlets. This process is repeated for each chip, on each injection iteration, thus showing the overall trend of the resistance as a function of injection number for these devices. Different colors indicate different comb chips. (**B**) shows the location of the segments where the electrical measurements are taken.

The set of 10 comb chips have a total of 320 outlet channels. It is impractical to measure the resistance of each channel after each CNF iteration. Additionally, measuring each channel is unnecessary, as the purpose of intermediate measurements is to track the progression of the wiring, so representative subsets are sufficient. The chips can be conceptually broken into four segments as defined by the second branching event, where the segments are made up of channels 1–8, 9–16, 17–24, and 25–32, respectively, as shown in Figure 4B. Every measurement taken has one lead inserted into the common inlet port (labeled `top port’ in Figure 4B) and the other lead inserted in one of the 32 outlet ports. For each of the four measurements taken between CNF iterations, the other lead is inserted in a randomly chosen channel in the corresponding segment. The average of the four measurements taken per chip is plotted for each comb chip as a function of injection cycle count in Figure 4A. All electrical measurements are taken when the channel is dry. Figure 4A shows convergence towards a saturated resistance value on the order of tens of kΩ, along with a reduction in variation between individual chips as the injection iteration number increases.

***Cold Room Setup***. In the area wherein the experiment is set to run, a header tank with a constant flow of seawater is continuously filled and connected to a PVC line. Figure 2 shows a photograph of the cold room setup. The PVC line has triple differential flow configurations spaced for each experiment, also made out of PVC and connected to vinyl tubing. These configurations allow the water flow for each individual MFC in the experiment to be adjusted without affecting the others so that the optimal amount of water is constantly supplied. Just behind the water flow PVC is another line identical in length that has individual holes, adjustable valves, as well as tubing. This ensures that each MFC also has adequate oxygen flow for the aerobically necessary cathodic reaction. The MFCs (inside their respective 1 L beakers) are placed inside a plastic tub, 3 at a time, which fits them and separates the experiment properly. The tubes for water and oxygen are clamped to the beaker so that they are slightly penetrating the water surface. The experiment is left to run, and the water flow/oxygen flow/separation of the MFC components within the beakers are tightly and constantly monitored.

***PBS and Acetate Solution, Anode Preparation, and Electronic Connections***. Acetate is often used as a bacterial food source, as it is a single carbon compound easily used as a food source by many exoelectrogens. A 50 mM phosphate buffer solution (PBS) is used to create a 1 g/L sodium acetate solution via dissolution with subsequent stirring. This is accomplished by adding 5 mL of sodium acetate solution to 5 mL of seawater and mixing. Next, 5 mL of 50 mM PBS is added to 5 mL of seawater and mixed. A total of six anodes are used—three anodes are injected with 2 mL of the PBS–acetate–seawater solution, and three anodes are injected with 2 mL of the PBS–seawater solution.

The microfluidic microbial fuel cell anodes are connected to a patented electrical regulation system which measures the open circuit voltage potential of the benthic microbial fuel cell (BMFC) between the anode and cathode for the first 3 days. This potentiostat then sets the appropriate load resistor value, in this case 10 kΩ, to maintain the voltage. Power is determined using current multiplied by operating voltage.

***MBMFC Construction***. To assess the applicability of the design, six MBMFC devices are tested at NIWC Pacific. All six anodes are crimped to titanium wire and are covered with marine grade heat shrink. Any exposed copper wire from the anodes is coated with clear nail polish to minimize corrosion. Approximately 500 mL of sediment is placed in a 1 L glass beaker. All six anodes are submerged into the sediment with only the top 1/4 of the anode plastic exposed. A carbon cloth cathode for each is sewn to copper wire, heatshrunk with marine grade heat shrink and is submerged just beneath the water surface. For the duration of the experiment, a steady flow of ocean salt water and aeration is supplied to each of the six anodes. Figure 3 shows a schematic of the experimental setup.

***Sediment Supernatant***. San Diego Bay sediment is obtained from the Marine Corp Recruit Depot located at 32°44′20.65″ N, 177°12′31.42″ W. Two 50 mL conical tubes are filled roughly three-quarters full with sediment slurry. The tubes are then centrifuged for 2 min at 500 RCF and then another 2 min at 700 RFC to pellet out the sediment/debris, leaving the bacterial inoculate in the supernatant. This supernatant is then decanted into another conical tube for the subsequent treatments.

## 3. Results and Discussion

### 3.1. Chip Design

**Figure 1:**
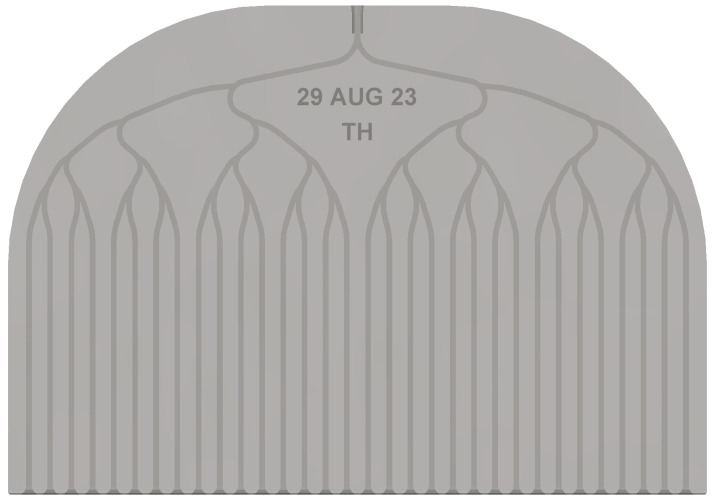
CAD representation of the comb chip design showing a front view. The non-uniform branching structure is designed to minimize fluidics resistance from sharp bends. Sharp corners can also produce low-velocity traps for particulates, which can increase clogging likelihood in practical devices, as well as increasing the overall fluidic resistance.

**Figure 2:**
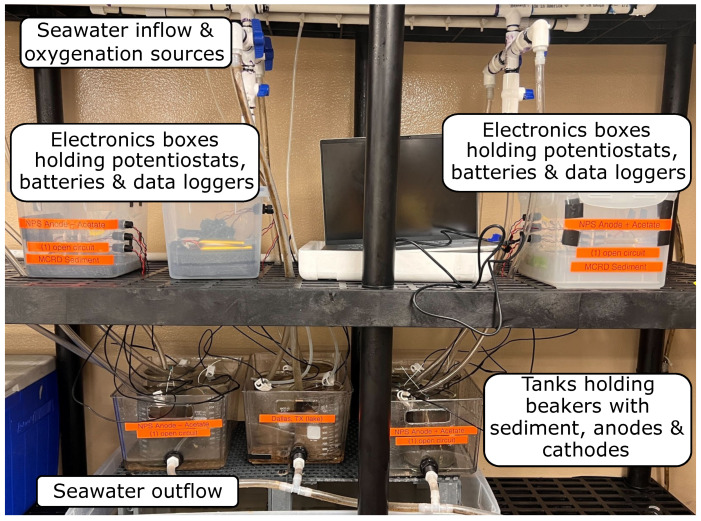
Labeled photograph of experimental setup in cold room seawater flow chamber at Naval Information Warfare Center Pacific, San Diego.

**Figure 3:**
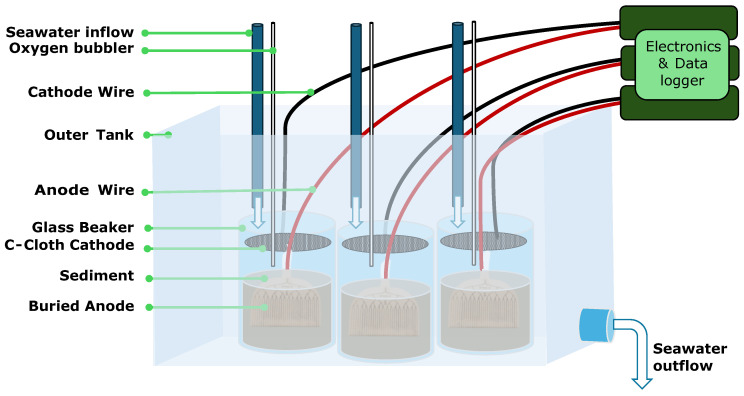
Diagram of one tank of experimental set-up. Cathodes are implanted in sediment with a cathode in the overlaying water column. Each experiment is connected to an individual electrically isolated regulating electronics and data collector.

To minimize the cumulative fluidic resistance of the comb chip’s microchannels, the branching connections are designed to avoid sharp changes in direction. This results in the streamlined and naturalistic appearance of the network of microfluidic channels. The channel arclengths are non-uniform (see Figure 1). As measured from the channel’s centerline in SOLIDWORKS, the set of arclengths contains a maximum of 100.89 mm, a minimum of 86.44 mm, and a mean of 93.14 mm. Additional features include the outlet ports being designed to minimize head loss for the internal flow, an increase in size near the inlet to allow for easier processing and wiring, and a robust minimum spacing of 2.23 mm between channels.

### 3.2. Wiring Procedure

**Figure 5:**
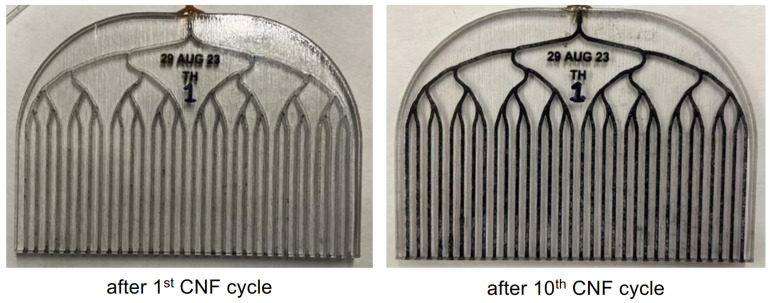
Visualization of CNF deposition inside the microchannels of comb chips. As the injection is iterated, each time, more CNFs are deposited throughout the volume of the network of microfluidic channels. This accumulation effect can be seen as a darkening of the microfluidic networks.

After fabrication and support material removal, the chip’s capturing electrode is created via CNF deposition [54]. The experimental chips are subjected to 10 CNF injection cycles, resulting in a functional distributed electrode. The development of the wiring can be visually seen in Figure 5. Additional details on this process can be found in the relevant subsection of the Materials and Methods Section.

The decision to use CNFs is made due to the electrical conductivity and biocompatibility of the CNFs, and the porosity of the resulting CNF matrix. Electrical conductivity is critical, as it allows the electrons to be captured and thus allows current to flow and electrical power to be extracted. Biocompatibility ensures that the CNF material is not toxic to the bacteria and allows the exoelectrogenic microorganisms to have a readily available terminal electron acceptor within microscopic distance to capture the electrons. The CNF matrix porosity allows the bacteria to move about throughout the wiring without being impeded. Porosity also allows for fluidic throughput, which is necessary to get the bacteria inside the microchannels and cycle them out if desired. Porosity is also what allows for the bacteria to be effectively inside the wiring, thus further decreasing the distance from electron emission to electron capture. This combination of features should contribute to the further improvements of the capture efficiency compared to previous 2D results [49] and make more efficient use of the internal chip volume.

### 3.3. Electrical Characterization Results

Understanding the electrical properties of the capturing electrode is highly important for the development of these devices. As such, electrical characterization is performed on the dry, wired chips before the introduction of bacteria. Figure 5 shows qualitative visible indications of progress with the CNF injections. Figure 4A indicates the same progress quantitatively in terms of measured resistance decreasing with injection number.

As expected, the electrical resistance decreases with the deposition of more carbon to the channels. This results in resistances on the order of tens of kΩ after 10 injection cycles. Representative resistance measurements are recorded after each injection cycle of each chip. The individual data points shown in Figure 4A are each an average of the four representative measurements taken on each comb chip. Figure 4A shows a clear downward trend in resistance, indicating improvement in the conductivity of the wiring that spans several orders of magnitude.

**Figure 6:**
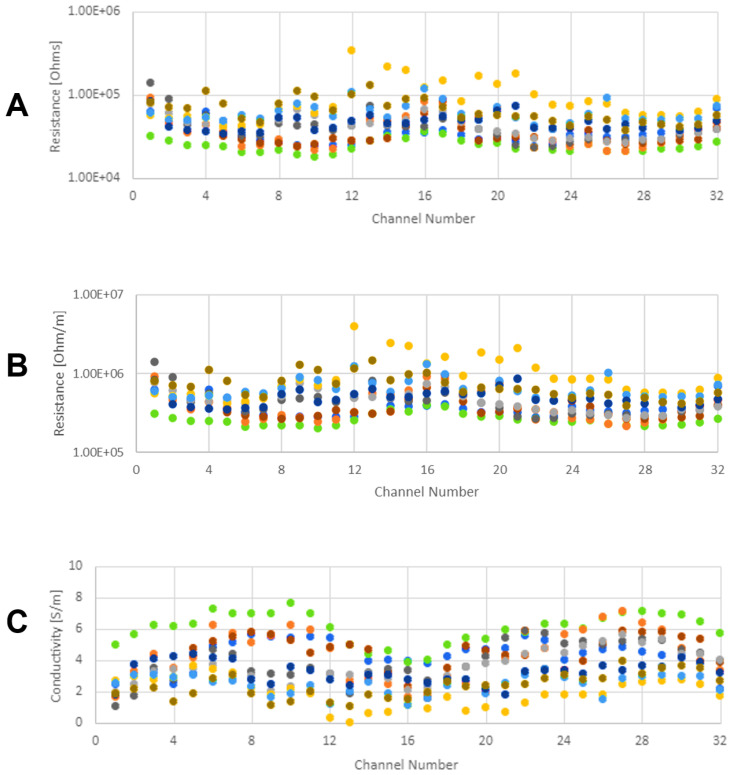
Characterization of electrical properties of the finished test chips. (**A**) shows the resistance of each separate channel. These resistance values are measured between the channel’s individual outlet to common inlet. The final resistance of individual channels is plotted against the respective channel ID number, while color identifies the respective chip. The same data from (**A**) are normalized with respect to channel arclength, resulting in (**B**) showing channel resistance per unit length plotted against the respective channel ID number, while color identifies the respective chip. This arclength normalized data in conjunction with cross-section data are used to calculate effective conductivity for each channel as shown in (**C**). (**C**) shows channel conductivity plotted against the respective channel ID number, while color identifies the respective chip. Different colors indicate different comb chips.

**Figure 7:**
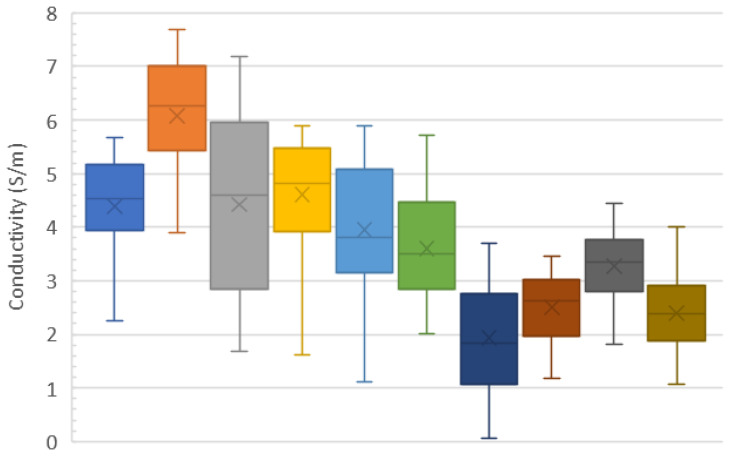
Average electrical conductivity of each comb test chip. Box-and-whisker plots along with a superimposed “x” showing the mean value for each device show how uniform the wiring process is. By convention, the ends of each “whisker” correspond to the extreme values of the data set, the edges of the “box” correspond to the first and third quartile, respectively, and the line in the middle of the “box” represents the median. Different colors indicate different comb chips.

Once the wiring process is complete, more thorough characterization is performed as shown in Figure 6 and Figure 7. Measurements are taken of each channel on each chip (as opposed to four representative measurements per chip used for intermediate measurements) and then plotted as a function of the channel number (Figure 6A). Since the channels do not share a uniform arclength, it is necessary to normalize the raw resistance (shown in Figure 4A and Figure 6A) to resistance per unit length (shown in Figure 6B) by dividing the former by the length of the channel being measured. The arclength-normalized resistance is then inverted to calculate the conductivity of the microchannels (Figure 6C). The final conductivity of each comb chip is shown with box-and-whisker plots (Figure 7), wherein an additional x shows the location of the mean.

The CNF matrix is a 3D electrode distributed through the volume of the microchannel, whereas previous microfluidic work [49] limited the electrode to a 2D substrate. As a result, the new system boasts a significantly reduced mean distance between any given bacterium and the closest point on the electrode. Previously decreasing this mean distance resulted in a 4× power density increase compared to earlier devices [49]. It stands to reason that the 3D CNF distributed electrode could in principle further increase the resulting power density even when compared to the 2D microfluidic approach [49], while allowing for practical upscalability.

### 3.4. Electrical Characterization Considerations

The distributed electrode importantly allows the emitted electrons to be captured efficiently independent of location by providing a path for the current to flow that is adjacent to the point they are emitted, with minimal impedance of fluidic throughput:

(1) $$\begin{array}{cc} R_{\mathrm{wet}} & =\frac{R_{W}\cdot R_{\mathrm{CNF}}}{R_{W}+R_{\mathrm{CNF}}} \end{array}$$

(2) $$\begin{array}{cc} R_{\mathrm{dry}} & =\frac{R_{\mathrm{air}}\cdot R_{\mathrm{CNF}}}{R_{\mathrm{air}}+R_{\mathrm{CNF}}} \end{array}$$

(3) $$\begin{array}{cc} R_{\mathrm{wet}} & ≅\frac{R_{W}\cdot R_{\mathrm{dry}}}{R_{W}+R_{\mathrm{dry}}} \end{array}$$

All electrical measurements are taken when the channel is dry; thus, they represent the combined conductivity of the CNF wiring and the air in the channel (see Equation (2)). Because air is an electrical insulator, the measured overall dry conductivity is due to the conductivity of the CNF wiring. When the air is replaced with seawater, the overall conductivity of the channels is expected to increase roughly as suggested by the relationship shown in the equation. Equations (1)–(3) are based on the idea that the distinct materials in a microchannels electrical behave like resistors connected in parallel.

When seawater containing bacteria is loaded inside the channels, the seawater produces new conductive pathways. These added pathways introduce new paths of least resistance in the CNF network. Thus, the conductivity is enhanced beyond the prediction made by Equation (1), as they provide additional electrical pathways along the matrix structure as well as functioning as parallel resistors. This is generally beneficial. If it is assumed that there is no electrical interaction in the form of new pathways between the seawater and CNF wiring, then the combined resistance of the wet channel can be calculated with the parallel resistor model shown in Equation (3):

(4) $$R_{\mathrm{dry}}≅R_{\mathrm{CNF}}$$

In the equations, $R_{W}$ represents the resistance of the seawater in the channel. $R_{\mathrm{wet}}$ represents the total resistance of the channel once seawater is loaded alongside the CNF wiring. $R_{\mathrm{dry}}$ represents the empirically measured resistance of the CNF wired channel with air as the fluid surrounding the wiring. $R_{\mathrm{air}}$ represents the resistance of the air inside the channel along with the CNFs. $R_{\mathrm{CNF}}$ represents the theoretical resistance of only the distributed CNF wiring ignoring the effects of air. $R_{\mathrm{CNF}}$ can be approximated as $R_{\mathrm{dry}}$ by applying the condition that $R_{\mathrm{air}}\gg R_{\mathrm{CNF}}$ to Equation (2). Thus, $R_{\mathrm{CNF}}$ as defined in Equation (1) can be approximated as the resistance of the dry channel due to the negligible effect the air will have. This results in the relations described by Equation (3).

If the initial assumption (that the seawater and the CNF wiring will conduct current fully independently of each other, in other words no new electrical pathways are formed) is not satisfied, $R_{\mathrm{wet}}$ will be lower than Equation (3) predicts. This decrease could be due to the newly available paths of least resistance available by the change in breakdown voltage due to the change in fluid. Thus, $R_{\mathrm{wet}}$ as calculated by the parallel resistor model described by Equation (3) can be thought of as an upper limit for the actual resistance of the channel when in use, thus forming a conservative estimate.

Using seawater as part of the conducting network could produce some electron loss and thus power loss due to local recombination. However, if this is performed over very short distances (like the distance from the bacteria to the nearest portion of the CNF wiring), the recombination probability is expected to be low. The overall result should be an even more efficient capture matrix than predicted by the measurements taken when the channel is dry as described by Equation (2) and plotted in Figure 4, Figure 6 and Figure 7. Thus, even higher capture efficiency, and thus even higher output power density, can be expected.

### 3.5. MBMFC Device Overall Performance

**Figure 8:**
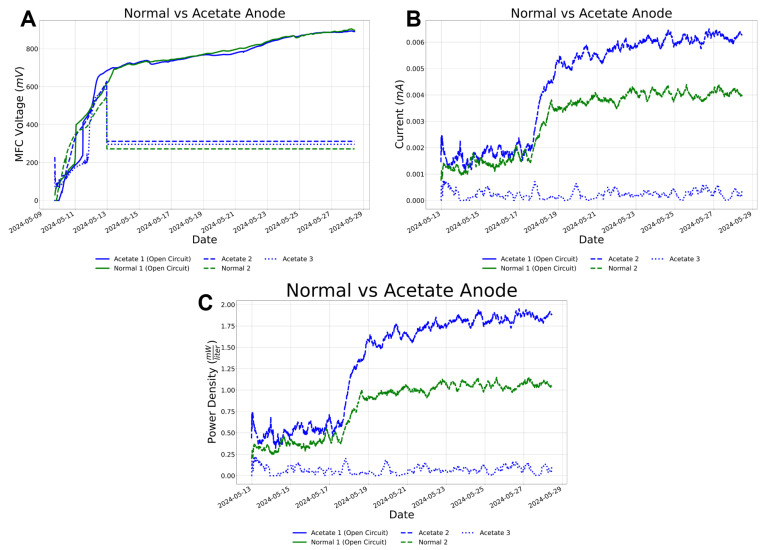
Results of the test and evaluation of six MBMFC devices at NIWC Pacific. (**A**) MBMFC voltage over time. (**B**) MBMFC current over time. (**C**) MBMFC power density over time.

Of the 10 MBMFC devices, 4 were rejected at the last minute due to clogged inlet ports. Therefore, six were selected to determine energy generation by indigenous bacteria from sediment. MBMFC whole cell potential and current were sampled at 10 min intervals during the laboratory experiments from 13 May 2024 to 28 May 2024 (Figure 8). Upon being placed in beakers, load potentials from five MBMFC devices began to rise within one day, demonstrating that bacteria were present and indicating that the comb chip’s microfluidic networks were becoming anaerobic. One MBMFC device failed to function for unknown reasons. Two MBMFC units were maintained at open circuit to serve as a control for the units to ensure that anaerobic conditions are possible within the units and that clogging does not occur. Acetate was added to one of the open circuit units (Acetate 1-Open Circuit), whereas Normal 1-Open Circuit served as the units where only a sediment slurry was added without the acetate as an additional food source.

In Figure 8A, the load of the open circuit units rises to ≈900 mV within this time-frame, showing that the units remained under anaerobic conditions. The other three tested units, Acetate 2, Acetate 3, and Normal 2 voltage, are dropped to ≈300 mV to generate power and maintained over the two-week experiment (Figure 8A), suggesting the formation of microbial exoelectrogens within the MBMFC anodes capable of donating electrons for power generation. Over the two weeks, each MBMFC maintains a whole cell potential at or above the preset 350 mV potentiostat discharge potential. The current generated (Figure 8B) is normalized to power density, which shows $\mathrm{mW}\;L^{-1}$, to allow for future comparisons between other 3D devices. Figure 8B,C show that Acetate 3 does not generate much power (on the order of $0.15\;\mathrm{mW}\;L^{-1}$) and this is attributed to one of the used combs having higher internal biological resistance. However, when examining the Acetate 2 versus Normal 2 units, there is a greater difference in power generation: $\approx 1\;\mathrm{mW}\;L^{-1}$ versus $\approx 2\;\mathrm{mW}\;L^{-1}$. This shows that there is a great potential for these comb chips and power generation, especially when a food source is added.

Three MBMFC devices, through harnessing the metabolic activity of microorganisms, efficiently converted organic matter into electrical current (Figure 8B), demonstrating the MBMFC device’s potential as a renewable energy source. The cell’s electrodes facilitated the electron transfer process, enabling the microorganisms to oxidize organic compounds and generate electrical current. Notably, the MBMFC exhibited a powerful potential for electricity generation. Its eco-friendly nature, coupled with its ability to operate in diverse environmental conditions, underscores its promise as a viable solution for generating clean energy.

## 4. Conclusions

This work shows the potential of MBMFC for practical upscaling. Indeed, there are needed improvements and optimizations that remain. These include improvements to the wiring technique, fabrication process, modularity, microfludic network geometry and electrical attachment methods. At present, these units can be fabricated on the order of weeks using less human labor versus the typical assembly of BMFCs. Although the tests produce low power densities, this is still considered to be a significant improvement, as the ability to upscale is critical. With this characteristic met, true optimization work can begin. Additionally, forthcoming improvements should increase the output currents and thus the output power density. This opens up opportunities for these units to be connected as modules to achieve the increased power production needed to support remote sensors in small form factor battery recharging stations and other applications.

## Data Availability

All materials, data and associated protocols contained in this manuscript can be made available to readers upon request.

## Abbreviations

STL	Standard Tessellation Language
CAD	Computer-Aided Design
